# Clinical significance of unexplained persistent sinus tachycardia in women with structurally normal heart during the peripartum period

**DOI:** 10.1186/s12884-022-05012-3

**Published:** 2022-09-03

**Authors:** Dimitrios Varrias, Nikhil Sharma, Roland Hentz, Rosaline Ma, Dillon Gurciullo, Jeremy Kleiman, Andrew Kossack, Eliot Wolf, Betty Lam, Tia Bimal, Umair Ansari, Kristie M. Coleman, Stavros E. Mountantonakis

**Affiliations:** 1 Department of Cardiology, Lenox Hill Hospital Heart & Lung, Northwell Health System, 100 East 77th Street, 2nd Floor, New York, NY 10075 USA; 2 Feinstein Institutes For Medical Research, Manhasset, NY USA; 3 Department of Obstetrics & Gynecology, Lenox Hill Hospital, Northwell Health System, New York, NY USA

**Keywords:** Persistent sinus tachycardia, Peripartum period, Arrythmia management during pregnancy, Cardio-obstetrics

## Abstract

**Background:**

Persistent sinus tachycardia (ST) is frequently encountered during pregnancy and peripartum period and its etiology often remains elusive. We sought to examine the possible association between unexplained persistent ST and obstetric outcomes.

**Methods:**

A case control study was conducted using chart review of women admitted in labor to one of 7 hospitals of Northwell Health between January 2015 to June 2021. After excluding women with structurally abnormal hearts, we identified patients with persistent ST during the peripartum period, defined as a heart rate of more than 100 bpm for more than 48 h. A control group was created by randomly subsampling those who did not meet the inclusion criteria for sinus tachycardia. Obstetric outcomes were measured as mother’s length of stay (LOS), pre-term labor (PTL), admission to the neonatal ICU (NICU), and whether she received cesarean-section (CS).

**Results:**

Seventy-eight patients with persistent ST were identified, out of 141,769 women admitted for labor throughout the Northwell Health system. 23 patients with ST attributable to infection or hypovolemia from anemia requiring transfusion and 55 with unclear etiology were identified. After adjusting for age and parity, pregnant mothers with ST were 2.35 times more likely to have a CS than those without (95% CI: 1.46–3.81, *p* = 0.0005) and had 1.38 times the LOS (1.21- 1.56, *p* < 0.0001). Among mothers with ST, those with unexplained ST were 2.14 times more likely to have a CS (1.22–3.75, *p* = 0.008).

**Conclusion:**

Among pregnant patients, patients with ST have higher rates of CS.This association is unclear, however potential mechanisms include catecholamine surge, indolent infection, hormonal fluctuations, and medications. More studies are needed to explore the mechanism of ST in pregnant woman to determine the clinical significance and appropriate management.

**Supplementary Information:**

The online version contains supplementary material available at 10.1186/s12884-022-05012-3.

## Introduction

Cardiovascular complications are the leading non-obstetric cause of maternal morbidity and mortality during pregnancy [1]. Adverse maternal outcomes have been well described in women with structural heart disease and have been attributed to the pre-existing cardiac conditions, as well as advanced maternal age [2–5]. Physiologic changes during pregnancy predispose women to arrhythmias, notably supraventricular tachycardia, atrial fibrillation, and ventricular arrhythmias and women with arrhythmias during pregnancy have greater incidence of in-hospital death as well as maternal or fetal complications [6] especially for those women with underlying heart disease [7–9].

Sinus tachycardia (ST) is often encountered in women in pregnancy and particularly in labor. Although ST is often secondary to hypovolemia, hypoxemia, anemia or infection; it commonly occurs without an identifiable pathologic condition. Unexplained ST is generally believed to be a benign finding, representing physiologic response to neurohormonal changes, volume distribution, anxiety and pain. As such, reassurance and conservative management is the recommended management for unexplained ST [10]. Nevertheless, the association between unexplained ST with obstetric outcomes has not been examined. Through our analysis, we investigate the prevalence and etiology of persistent ST in a large cohort of women with structurally normal heart admitted in labor and explore association with adverse maternal outcomes.

## Methods

### Study population

We searched our common electronical medical record (EMR) to identify all women admitted in labor from January 2015 to June 2021 to any of the 7 Northwell Health Hospitals. We included the first admission to a Northwell Health facility for every mother that concluded in delivery.

Multiple gestational pregnancies were included. This study received exempt approval from the Northwell Human Research Protection Program (20–1064 LHH). The study received a waiver of consent and HIPAA authorization from Northwell Human Research Protection Program. Due to this waiver of consent from the Northwell HRPP consent did not have to be obtained.

Data extraction was performed during a detailed EMR search of all medical notes, diagnoses, medications, orders, vital signs and ECG & telemetry interpretations. We excluded patients with diagnoses of cardiomyopathy, coronary artery disease, ischemic heart disease, myocarditis, valvular disease, congenital heart disease, rheumatic heart disease, atrial fibrillation and presence of a cardiac device. We also excluded women with hyperthyroidism. Patients with structural heart disease, as identified by ICD codes were excluded from the study during the initial EMR query. Additionally, the records of patients included in the final analysis were manually adjudicated for the presence of structural heart disease.

The medical records identified by the data query were reviewed manually and adjudicated for the presence of a newly diagnosed arrhythmic event during hospitalization. While all newly diagnosed arrhythmic events were identified and included in the arrhythmia cohort for data collection, we chose to focus this analysis on women with sinus tachycardia. To identify patients with ST, records were scanned for keywords such as “tachycardia”, and “sinus tachycardia”. Additionally, all available vital signs and ECG were reviewed for presence of ventricular rate of above 100 bpm. Our study group consisted of patients with a persistent heart rate of more than 100 bpm during the admission and for at least 48 h. Charts of identified patients with possible ST were further adjudicated manually to confirm the diagnosis of persistent ST.

All patients with persistent ST had an internal medicine or cardiology consult, where possible the etiology of ST was documented. In the majority of patients, no identifiable complications were documented at the time the consult was initiated. We cannot rule out anxiety, however no signs of hypovolemia, infection, extreme pain, hyperthyroidism or pulmonary embolism were identified in patients where etiology was classified as unknown.

**Fig. 1:**
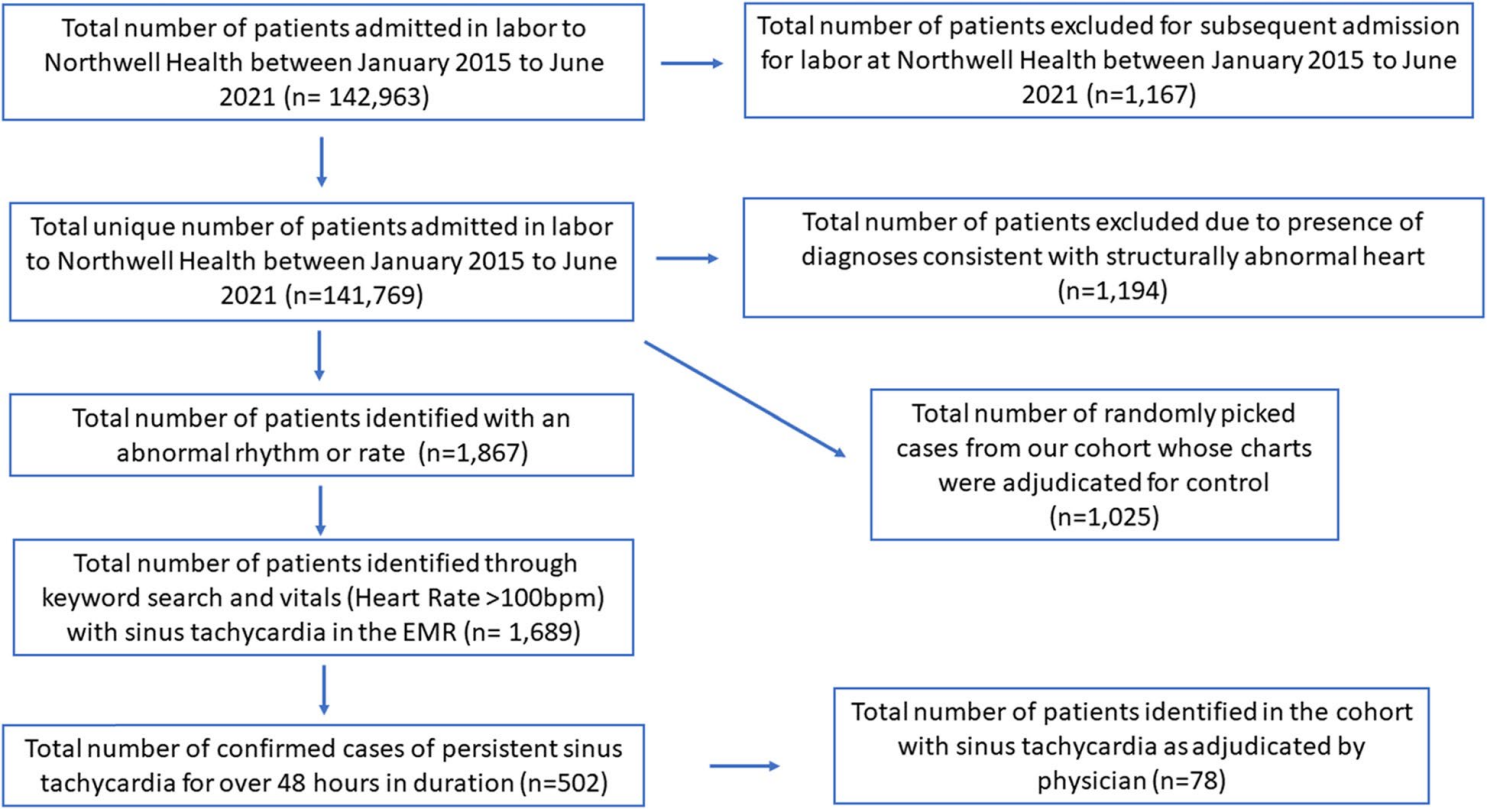
Eligibility Criteria for Study Cohort

A random set of control patients, with no history of arrhythmia or arrhythmia during the peri-labor period and structurally normal hearts, was selected by sub-sampling the available set of patients without arrhythmias (Fig. 1).

### Clinical outcomes

Outcomes including maternal mortality, Cesarean section, pre-term birth (< 37 weeks of gestation), mean length of stay (LOS) and infant requiring care in the neonatal intensive care unit (NICU) were evaluated between the ST and the control group. Infant requiring care in the neonatal intensive care unit was considered as a clinical outcome but was excluded from some analyses due to low frequency counts. No maternal or fetal mortality was noted in the cohort.

### Statistical analysis

The proportion of patients with sinus tachycardia out of the total number of patients screened was calculated. Normally distributed data are presented as mean ± SD and non-normal data as median (interquartile range [IQR]). Categorical data are presented as frequency (percentage of the total).

Association between ST and pre-term birth and C-section were tested using multiple logistic regression (modeled separately). Association between ST and hospital length of stay were tested using negative-binomial regression. Association between ST in the peri-labor period and NICU admission were tested using a Fisher’s exact test.

Study data were collected and managed using REDCap electronic data capture tools hosted at Northwell Health. The data analysis for this paper was generated using SAS Version 9.4 (Cary, NC, USA).

## Results

### Patient characteristics

Of 142,963 unique patients admitted in labor between January 2015 to June 2021, we excluded 1,194 due to presence of diagnoses consistent with structurally abnormal heart. Keyword search for patients with arrhythmic events in the EMR yielded 1,867 patients. During manual adjudication 1 control and 2 arrhythmia patients were removed due to presence of structural heart disease.

Sinus tachycardia that fulfilled the inclusion criteria were met in 78 (0.055%) of 141,769 patients. The control group included 1025 patients sub-sampled from the cohort of 141,769 patients. Clinical and demographic characteristics of the patient population are listed in Table 1 for both the sinus tachycardia and control cohorts. Study and control groups had similar age, parity and comorbidities except hypertension that was more prevalent in the arrhythmia group (9.0% vs 4.0%) (Table 1). In our cohort 8 women in the ST group carried the diagnosis of preeclampsia, 2 of them were diagnosed with infection and the tachycardia was attributed to this. In the remaining 6 women with preeclampsia and sinus tachycardia, their persistent tachycardia was labelled as inappropriate.

**Table 1:** Baseline Characteristics

	Sinus Tachycardia (*N* = 78)	Control (*N* = 1025)
Demographics		
Age	30.38 ± 5.34^a^	31.37 ± 5.43^a^
Race		
White	28 (35.9)	499 (48.7)
African American/ Black	15 (19.2)	116 (11.3)
Asian	14 (18.0)	97 (9.5)
Native American/Alaskan	1 (1.3)	10 (1.0)
Native Hawaiian/Pacific Islander	0 (0)	1 (0.10)
Multiracial/Other	14 (18.0)	241 (23.5)
Unknown	6 (7.7)	61 (6.0)
Comorbidities		
Hypertension	7 (9.0)	41 (4.0)
Diabetes	0 (0)	11 (1.1)
Chronic Kidney Disease	0 (0)	1 (0.10)
Chronic Obstructive Pulmonary Disease	0 (0)	1 (0.10)
Parity	1.0 (1.0–2.0)^b^	2.0 (1.0–2.0)^b^
Multiple Gestational Pregnancy	2 (2.6)	20 (2.0)

^a^ Mean ± Standard deviation

^b^ Median (1^st^ Quartile-3^rd^ Quartile)

N (%) unless otherwise specified

Note: There was no statistically significant difference noted between the sinus tachycardia and controls for baseline characteristics

All of the patients in the cohort presented with ST, of the ST cohort, 58.9% were discharged in ST. Vital signs were recorded every 6 h per protocol and more frequently when indicted. The average HR upon presentation was 111.8 ± 10.7, while the average HR upon discharge was 98.4 ± 13.1.

#### Etiology of sinus tachycardia

**Fig. 2:**
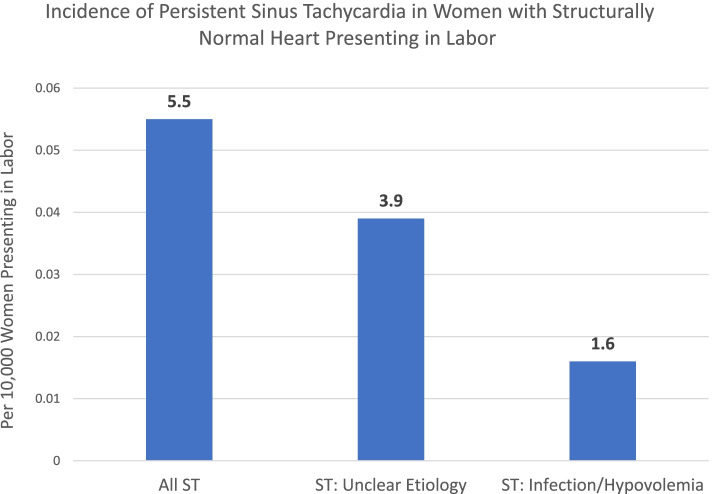
Incidence of Persistent Sinus Tachycardia in Women with Structurally Normal Heart Presenting in Labor

Out of 78 women with persistent ST, 10 were found to be hypovolemic due to blood loss, 13 had an infection requiring antibiotic treatment but the remaining 55 were tachycardic without an identified etiology (Fig. 2).

#### Clinical events and outcomes

The odds ratios of the specified clinical outcomes for the sinus tachycardia group, stratified by etiology compared to the control are shown in Table 2.

**Table 2:** Odds Ratio for Adverse Maternal Outcomes by Sinus Tachycardia Etiology

Outcomes	Sinus Tachycardia (*N* = 78)	Unclear Etiology (*N* = 55)	Hypovolemia/Infection (*N* = 23)
C-Section	2.35 (1.46–3.81)	2.14 (1.22–3.75)	2.97 (1.25–7.10)
Length of Stay^a^	1.38 (1.21–1.56)	1.16 (0.99–1.36)	1.89 (1.54–2.31)
NICU	13.3 (0.82–214.7)		
Pre Term	0.83 (0.32–2.13)	0.95 (0.33–2.72)	0.55 (0.07–4.19)

Odds ratio (95% Confidence Interval)

^a^ Mean length of stay, presented as mean ratio

#### Cesarean-section

There were 44 (56.4%) patients who underwent CS in the ST group and 364 (35.5%) in the control group.

When adjusted for age and parity, women with persistent ST during the peripartum period were 2.35 times more likely to undergo CS (95% CI: 1.46–3.81, *p* = 0.0005) than control patients.

Among women with ST with identified etiology, 14 (60.8%) underwent CS, while 30 (54.6%) with unclear etiology underwent CS. When clustered by etiology, patients with sinus tachycardia secondary to an identified etiology (infection or hypovolemia) had 2.97 times the odds (1.25–7.10, *p* = 0.0142) of undergoing a CS, while in patients with an unclear etiology were 2.14 times more likely to undergo CS (1.22–3.75, *p* = 0.008) than a control patient.

The indication for CS in patients in the ST group was non-reassuring fetal heart tracing (8), arrest of descent (6), arrest of dilation (5), nuchal cord complication (4), unclear (4), tachycardia (4), preeclampsia (3), multiple gestations (3), chorioamnionitis (3), breech position (1), oligohydramnios (1), placenta previa (1) and preterm labor (1). There were no discernable trends in the indication for CS in the ST of unclear etiology group as compared to those with identified etiology.

#### Length of stay

Patients with ST during the peripartum period, as well as those in the control group, had a median length of stay of 3 days. Women with persistent ST in the peripartum period had 1.38 times the length of stay than patients without (1.21–1.56, *p* < 0.001).

When clustered by etiology, sinus tachycardia with infection or hypovolemia had 1.89 times the length of stay compared to those without (1.54- 2.31, *p* < 0.0001), while patients with an unclear etiology had 1.16 times the length of stay, although this association was not statistically significant (0.99–1.36, *p* = 0.069).

## Discussion

With this analysis we found persistent sinus tachycardia during the peripartum period was significantly associated with increased length of stay and c-section. The etiology of persistent ST was attributable to infection or hypovolemia due to blood loss in the minority of cases (29.5%) while the etiology was unclear for the remainder of the cohort (70.5%). Both categories were found to be associated with increased rates of c-section, while only those with infection or hypovolemia had lengthier hospital stays. The focus of our analysis was on the primary outcome of c-section, it is not surprising those who had a c-section also had secondary outcomes of hypovolemia or infection and therefore a prolonged length of stay. It is important to identify occult reasons for sinus tachycardia such as hypovolemia or infection, as was the case in 29.5% of our cohort. However, these findings suggest there is poor understanding of the etiology of sinus tachycardia, and its clinical significance during the peripartum period may be often underestimated.

During pregnancy, systemic and pulmonary vascular resistance are decreased through the release of progesterone and estrogen to allow for increased maternal cardiac output. Stroke volume increases during the first semester through increased cardiac preload facilitated by the renin–angiotensin–aldosterone system [11]. Increased heart rate mediated by the parasympathetic and sympathetic nervous system becomes the compensatory mechanism during the second and third trimesters for increased cardiac output [12]. In addition to plasma volume expansion, there is an increase in red blood cell production up to 40% via erythropoiesis. Plasma volume increases proportionally more than the red blood cell mass, resulting in a “physiological anemia” from hemodilution, with hemoglobin levels as low as 11 g/dL considered physiological [11]. Distinguishing between physiologic or pathologic sinus tachycardia due to these inherent alterations in the cardiovascular system of pregnant women can be challenging [13]. Fluctuations in normal physiological parameters makes determining a heart rate threshold to diagnose persistent sinus tachycardia difficult for even experienced clinicians [14]. Clinicians may be unaware that epidemiological studies have demonstrated that the expected median heart rate during pregnancy is 91 bpm (3^rd^ centile 68 to 97^th^ centile 115) at 34.1 weeks. Heart rates over 105 bpm were only seen in 10% of the 1041 women included in the study after 28 weeks. The American College of Obstetricians and Gynecologists recommends investigating heart rates over 115 bpm or over 110 bpm in conjunction with other findings or symptoms [15]. These heart rates are known to vary across diverse populations. Additionally, Savu’s et al. analysis of the morphological and functional adaptation of the maternal heart during pregnancy found maximum heart rate rarely exceed 100bpm [16].

Sinus tachycardia is generally considered a benign arrhythmia, subsequently management recommendations are absent from newly published guidelines [17]. Therefore, the question remains: if persistent sinus tachycardia is considered a benign occurrence, with the only intervention to the patient being reassurance [18], why do women with persistent ST of unclear etiology experience higher rates of c-sections Answering this question may be of vital significance as it may shorten then LOS but also prevent unnecessary C-sections which can be associated with short- and long-term effects for both mother and child [19].

In almost 70% of the patients in our cohort, the etiology of persistent ST could not be elucidated, and could be deemed as either inappropriate tachycardia or sinus tachycardia of unknown etiology. Inappropriate sinus tachycardia, defined by the 2015 HRS consensus as “sinus heart rate more than 100 bpm at rest with mean 24‐hour heart rate more than 90 bpm, in absence of secondary causes”, has been shown to predominately affect young females [20]. Shabtae et al. found pregnancy to be the most common precipitating factor for inappropriate ST [21]. Dysregulation of the compensatory mechanisms to increase cardiac output, namely sympathetic and renin–angiotensin–aldosterone system activation create autonomic dysfunction, is a known etiology for inappropriate ST [22, 23]. Only small case series in the literature report women who experience inappropriate ST during the peripartum may be at risk for adverse maternal outcomes. Sharp et al., found an increased rate of induction of labor among the 19 patients they identified, although they did not find elevated rates of c-section in this study conducted in the England [24, 25].

As shown in our study, sinus tachycardia cannot be explained solely by indolent infection or hypovolemia. Autonomic dysfunction, hormonal fluctuations, catecholamine surge, and medications have been proposed as explanations for sinus tachycardia of unknown etiology [26, 27]. Although clinicians must balance between extensive diagnostic investigation to determine the etiology of inappropriate ST and reassurance when it is a benign incident, reflecting physiologic changes, distinguishing between the two may have an impact on preventing adverse maternal outcomes. Further evidence is needed to understand the clinical significance and to deliver evidence-based care.

Through this analysis we emphasize the importance of not dismissing ST in the obstetric population and seek to raise awareness of its significance in predicating complications and poor maternal outcomes. ST is one of the earliest and most insidious signs of a poor prognosis, however it is frequently considered “physiologic”, even in the absence of a clear etiology. ST could be a surrogate of underlying pathology leading to poor outcomes and should prompt further investigation in obstetric patients.

### Study limitation

Several retrospective analyses have demonstrated the association between arrhythmia occurrence and increased mortality; however, these analyses were performed on administrative databases with known limitations due to coding. While potential patients with sinus tachycardia were identified using key word search throughout the EMR, we additionally applied a filter for heart rate and manually adjudicated the records to confirm structurally normal hearts, the clinical diagnosis and etiology.

## Conclusion

Occult reasons for sinus tachycardia such as hypovolemia or infection were identified in approximately a third of our cohort. Among pregnant patients, patients with unexplained ST have higher rates of C-Sections. Although potential explanations for this trend include catecholamine surge, indolent infection, hormonal fluctuations, and medications, further studies are needed to determine the clinical significance of ST and help physicians distinguish between an innocent physiologic response and a potential threat for the mother and the fetus.

## Supplementary Information

**Additional file 1: Supplementary Table.** ICD9 and ICD10 Procedure codes used to identify exclusion criteria before manual confirmation.

## Data Availability

The datasets used and/or analyzed during the current study are available from the corresponding author on reasonable request.

## Abbreviations

ST: Sinus tachycardia
CS: Cesarean section
LOS: Length of stay
