# Supplementary material for: Clinical significance of unexplained persistent sinus tachycardia in women with structurally normal heart during the peripartum period
Source: BMC Pregnancy Childbirth. 2022 Sep 3;22:677. doi: 10.1186/s12884-022-05012-3 (PMC9440559; doi:10.1186/s12884-022-05012-3)
Supplement: Supplementary file 1 — Additional file 1: Supplementary Table. ICD9 and ICD10 Procedure codes used to identify exclusion criteria before manual confirmation. [file 12884_2022_5012_MOESM1_ESM.docx]

Supplementary Table:

ICD9 and ICD10 Procedure codes used to identify exclusion criteria before manual confirmation

| Diagnosis | ICD10 Code | ICD9 Code |
| --- | --- | --- |
| Cardiomyopathy | I42 | 425 |
| Coronary artery disease | I25 | **429.2** |
| Ischemic heart disease | I42 | 410-414 |
| Myocarditis | I51 | 429 |
| Valvular disease | I34, I35, I36, I37 | 394,395,396, 397 |
| Congenital heart disease | Q24 | 745,746,747 |
| Rheumatic heart disease | I09 | 398 |
| Atrial fibrillation | I48 | 427 |
| Presence of a cardiac device | Z95.0, Z95.810 | 37.80, 89.49 |
| Hyperthyroidism | E05 | 242.9 |
